# miRNAs and their putative roles in the development and progression of Parkinson's disease

**DOI:** 10.3389/fgene.2012.00315

**Published:** 2013-01-09

**Authors:** Garry Wong, Richard Nass

**Affiliations:** ^1^Department of Neurobiology, A.I. Virtanen Institute, University of Eastern FinlandKuopio, Finland; ^2^Associate Professor of Pharmacology and Toxicology, Indiana University School of MedicineIndianapolis, IN, USA

**Keywords:** alpha-synuclein, miR-7, neurodegeneration, dopamine

## Abstract

Small regulatory RNAs, such as miRNAs, are increasingly being recognized not only as regulators of developmental processes but contributors to pathological states. The number of miRNAs determined experimentally to be involved in Parkinson's disease (PD) development and progression is small and includes regulators of pathologic proteins, neurotrophic factors, and xenobiotic metabolizing enzymes. PD gene-association studies have also indicated miRNAs in the pathology. In this review, we present known miRNAs and their validated targets that contribute to PD development and progression. We also incorporate data mining methods to link additional miRNAs with non-experimentally validated targets and propose additional roles of miRNAs in neurodegenerative processes. Furthermore, we present the potential contribution of next-generation-sequencing approaches to elucidate mechanisms and etiology of PD through discovery of novel miRNAs and other non-coding RNA classes.

## Introduction

Parkinson's disease (PD) is an age-related neurological disorder with no known cure. It is the second most common neurodegenerative disease and affects 1–2% of the population. The disease is characterized by resting tremor, bradykinesia, and loss of dopaminergic neurons (reviewed in Nass and Prezedborski, 2008). The onset in sporadic cases generally occurs between 50 and 60 years of age, yet familial forms can occur at 40 years and younger. Treatment is typically given with the goal to replace dopamine (DA) loss through the precursor L-Dopa. Adjuncts to this therapy include the addition of pharmacologic agents that decrease the breakdown of DA such as cathechol-O-methyl-transfer inhibitors and DA agonists. More recent therapies include grafts of stem cells that replace DA cells and deep brain stimulation, (Arias-Carrión and Salama, 2011; Gibson et al., 2012; Palm et al., 2012; Weaver et al., 2012). Despite these advances, replacement therapy has remained the standard treatment for PD for nearly half a century.

The underlying etiology of PD remains elusive; however recent studies have implicated several genes in the DA neuron pathogenesis. α-synuclein (ASYN) has been identified as a major component of Lewy bodies, a morphological hallmark of PD (Spillantini et al., 1997). Genetic studies have also identified several mutations in ASYN as causative for early onset PD through a dominant mode of inheritance. Other genes found in human genetic studies linked to PD include: Parkin (PRKN), a ubiquitin ligase associated with the ubiquitin-proteosome system; DJ-1, a poorly characterized anti-oxidant protein; LRRK2, a putative protein serine threonine kinase; and PINK1, a putative PTEN induced protein kinase. PRKN is believed to target proteins for degradation in the proteosome through linkage of ubiquitin molecules (Tan and Lim, 2005). Mutations in PRKN cause a dysregulation of proteasome-associated protein degradation that contributes to DA neuron pathology. DJ-1 regulates oxidation–reduction signaling pathways via induction of gene expression (Bonifati et al., 2004). DJ-1 has been shown to inhibit DAergic cell death in a cell and animals models (Jeong et al., 2012; Sun et al., 2012). LRRK2 and PINK1 are both mitochondria kinases. LRRK2 mutations cause uncoupling of mitochondria in fibroblast and neuroblastoma cells (Papkovskaia et al., 2012). PINK-1 is believed to be involved in mitophagy (Pogson et al., 2011). LRRK2 and PINK-1 may also interact directly or functionally. Finally, at least 16 loci (PARK1-16) have been associated with the development of PD.

miRNAs are ~22 nucleotide non-coding RNAs processed by Drosha and Dicer from larger pri-miRNA (the initial transcript) and pre-miRNA (the 65–70 nucletide hairpin) (Lee et al., 2006; Ruby et al., 2006; Asikainen et al., 2007, 2008). miRNAs act to modulate transcription through the binding of mature miRNAs (~22 nucleotide processed form) to RNA-induced silencing complexes (RISC) followed by guidance to target cognate protein coding mRNAs. The miRNAs-RISC complex identifies target proteins coding mRNAs largely through identification of seed matches (sequences complementary to positions 2–8 of the miRNA) in the 3′ untranslated region. miRNAs can operate in tandem, cooperatively, or without an apparent seed sequence match. The miRNA-RISC complex then initiates a program for degradation or block of translation. Endogenous siRNAs are likewise effectors of gene silencing but are uniquely distinguishable from miRNAs by having separate biogenesis, amplification, silencing complexes, targeting, and mode of degradation (Lee et al., 2006; Ruby et al., 2006; Asikainen et al., 2007, 2008).

Several recent reviews have described a potential role that miRNAs may have in the development of PD (Gascon and Gao, 2012; Junn and Mouradian, 2012; Harraz et al., 2011; Mouradian, 2012). In this review we will focus on specific miRNAs and their relationship to PD. We also add studies from model systems to show the potential interplay between miRNA expression and DA neuron pathology.

## miRNAs in neuronal development

Early studies by Ambros and Horvitz defined a role of miRNAs in *C. elegans* development (Ambros and Horvitz, 1984). The investigators identified *lin-4* through *C. elegans* forward genetics as a small non-coding RNA involved in cell lineage defects. These studies were elaborated to include an axis of regulatory small RNAs and their target genes that include *lin-14* (Ambros, 1989). A large number of small RNAs were later cloned, and it was found that many were primarily expressed in the vertebrate brain (Lee and Ambros, 2001; Lagos-Quintana et al., 2002). That miRNAs might be involved in neuronal patterning was later established through another *C. elegans* forward genetic study in that identified *lsy-6* (lateral symmetry defective-6) as a miRNA necessary for left-right neuron asymmetry (Johnston and Hobert, 2003). These studies were extended to show that miR-273 acts sequentially and asymmetrically with *lsy-6* to confer left/right asymmetric expression of chemoreceptor genes in *C. elegans* neurons (Chang et al., 2004).

miRNAs also play a significant role in vertebrate neuron development (Sempere et al., 2004; Smirnova et al., 2005). In higher organisms, forward genetic studies are problematic due to the long generation times and for large animal population housing constraints. As a result, sophisticated and imaginative methods have been developed to identify the role of miRNAs in brain development. Kim and colleagues identified miR-133b as critical in midbrain DA neuron development (Kim et al., 2007). The investigators demonstrated a role for miRNAs *in vitro* by conditionally knocking out the Dicer enzyme through Cre-recombinase transduction in embryonic stem cells just prior to differentiation of the DA neurons (Kim et al., 2007). Cre-mediated deletion of Dicer at this stage resulted in nearly complete loss of DA neurons indicating the important role of Dicer processing of miRNAs (Kim et al., 2007). These findings were extended in the mouse nervous system using a Cre-recombinase under the regulation of a DA transporter that led to specific deletion of Dicer in post-mitotic midbrain DA neurons (Kim et al., 2007). These mice show loss of DAergic neurons and the behavior studies demonstrated reduced locomotion and immobility similar to human PD. To identify specific miRNAs that could underlie these affects, quantitative real-time PCR on a panel of 224 miRNAs were assayed and compared to miRNAs from PD and normal patients. miR-133b showed a significant deficiency in the midbrain PD relative to controls.

Consistent with the human study, miR-133b was reduced in two mice models of PD (Pitx3 deficient and 6-OHDA treated) (de Mena et al., 2010a,b). The authors propose that miR-133b acts with Pitx3 in a negative feedback circuit that regulates and maintains midbrain dopaminergic neurons. In order to highlight the critical role miRNAs play in neuronal specification, Yoo and colleagues recently reported the conversion of human fibroblasts into neurons using miR-9/9^*^ and miR-124 (Yoo et al., 2011). This effect was facilitated by NEUROD2. While the converted cells expressed neuron marker MAP2, and NMDA receptor 1, single cells did not express dopaminergic/noradrenergic markers TH or DDC. A broader perspective on the role of specific miRNAs in the discrete steps involved in neurogenesis was also recently described (Yang and Shi, 2012). Furthermore, miR-134 has been identified as a key regulator in development of dendritic spines in rat hippocampal neurons (Schratt et al., 2006).

## α-Synuclein confers DA neuron degeneration

ASYN protofibrils and fibrils accumulate and form protein aggregates in DA neurons and disrupt the proteosomal pathway. The protein aggregates result in declining cell function and eventually DA neuronal death leaving behind their Lewy body cores (Spillantini et al., 1997; Tofaris and Spillantini, 2007). The 3′ untranslated region of ASYN is under regulatory control of miR-7 that is predominantly expressed in neurons (Junn et al., 2009). miR-7 down-regulates ASYN protects against oxidative stress as shown *in vitro* and in mouse models, and normal levels are decreased in PD suggesting a role for miR-7 in PD pathogenesis (Junn et al., 2009). These findings were extended with the finding that miR-7 and miR-153 operate additively to reduce levels of ASYN in brain (Doxakis, 2010). A recent report shows that miR-7a also acts directly in controlling Pax6 in forebrain, which in turn specifies a DAergic neuron phenotype (de Chevigny et al., 2012). This is particularly relevant since it is now widely believed that neurogenesis occurs in postnatal and adult tissues in the mammalian forebrain. Here, neural stem cells separate spacially along the lateral wall of ventricles and the transcription factor Pax6 is an important determinant of cells demonstrating the DAergic cell characteristics. Theoretically, one therapeutic approach to treat PD may be to target ASYN through miRNAs. A “proof of concept” has already been demonstrated using a miRNA embedded AAV silencing vector that has been transduced in 293T cells and PC12 cells (Han et al., 2011). In this case, the miRNA was able to silence ectopically expressed ASYN suggesting that this approach is feasible. While these results are promising, future studies will need to address potential confounders such as the decrease sensitivity to RNA interference in neurons and in immune responses (Asikainen et al., 2005, 2007; Gehrke et al., 2010; Junn and Mouradian, 2012).

## Profiling miRNAs in PD tissues and PD models

In addition to the earlier study involving miR133b (Kim et al., 2007), other investigators have profiled miRNAs in PD tissues. Peripheral blood mononucleocytes from 19 patients and 13 controls revealed 18 differentially expressed miRNAs (Martins et al., 2011). The 622 predicted targets of 11/18 miRNAs were parlayed with Chip-seq data to converge upon ASYN protein, and the glycosphingolipid biosynthesis and ubiquitin proteosome pathways. A separate miRNA profiling study in human PD brain tissue of variable pathology found down-regulation of miR-34b/c. This misregulation was observed in both pre-clinical and clinical stages (Miñones-Moyano et al., 2011). *In vitro* follow-up studies that depleted miR-34b/c in SH-SY5Y cells differentiated to DAergic neurons found decreases in DJ-1 and PARK and altered mitochondrial function. In a separate study peripheral blood was obtained from eight untreated PD patients, seven early onset PD patients, and eight controls and profiled for 85 different miRNAs by quantitative real time PCR (Margis et al., 2011). miR-1, miR-22^*^, and miR-29 expression distinguished non-treated PD patients from health subjects, while miR-16-2^*^, miR-26a2^*^, and miR30a differentiated treated from untreated patients.

Consistent with the peripheral blood mononucleocyte study, whole genome microarray profiling of a *C. elegans* transgenic animals overexpressing human ASYN also identified genes in the uniquitin proteosome pathway that were dysregulated (Lakso et al., 2003; Vartiainen et al., 2006a,b). miRNA profiling of these transgenic animals also revealed the down-regulation of seven and the up-regulation of five miRNAs (Asikainen et al., 2010). *C. elegans* animals with a functional mutation in the vesicular monoamine transporter (VMAT; *cat-1* gene) that are unable to transport DA into synaptic vesicles were also profiled. The authors found two mRNAs were up-regulated and three down-regulated. Moreover, the PARK1 mutant *C. elegans* strain (*pdr-1*) was also profiled and two miRNAs were up-regulated while one was down. miR-64 and miR-65 were co-underexpressed in transgenic alphasyunuclein animals and *cat-1* strains. In the same study, *ptc-1* which encodes an ortholog of human PTCH, a sterol sensing domain patched gene, was confirmed as a target of these miRNAs. let-7 miRNA family members were also observed to be differentially expressed in human ASYN transgenic animals and PARK1 (*pdr-1*) mutants.

## PD genome-wide association studies (GWAS)

One of the earliest GWAS studies designed to identify miRNAs linked to PD was based on studies demonstrating that fibroblast growth factor 20 (FGF20) is associated with increased risk for developing PD (Moore et al., 2005). Using a sample size of 1089 affected and 1165 unaffected individuals, the strongest evidence of association was in a SNP located within the 3′ untranslated region of FGF20 (Wang et al., 2008). Functional assays were used to determine that the SNP disrupts a binding site for miR-433. Thus, the authors were able to identify in a cell system and PD brain that an increase in translation of FGF20 correlated with an increase in ASYN. The authors were also able to associate a SNP site that prevented miRNA activity with PD, and subsequent release of inhibition that was concomitant with increased expression of ASYN. This study provided a novel mechanism for susceptibility for PD. Unfortunately, linkage of the SNP in the 3′UTR of FGF20 could not be replicated in a Spanish population of 512 PD patients and 258 healthy controls (de Mena et al., 2010a) while the original study collected samples from PD centers in the U.S. In another study, a specific miR-133b homozygous variation could be found at a higher frequency in PD patients (6/575) than controls (0/650), but could not be confirmed in a second set of patients (1/250) vs. (2/210) controls (de Mena e tal., 2010b). Other GWAS studies have also been difficult to replicate: an association between polymorphism of miR-196a2 and PD could not be replicated in a Chinese population (Haixia et al., 2012); and variants found in the 3′ untranslated region of SNCA could not affect miR-433 binding (Schmitt et al., 2012).

## Bioinformatics of miRNAs in PD

Several bioinformatic tools have been used to integrate knowledge of miRNAs, their targets, and PD. One study has utilized miRNA target finders to associate key proteins involved in PD pathology such as ASYN, DJ-1, LRRK2, and PINK1 (Santosh et al., 2009). Variatas is a useful tool that allows for the retrieval of disease phenotypes and gene ontology data from public databases after entering miRNA-associated SNPs (Paananen et al., 2010). Utilizing another approach, miRo allows the user to enter a phenotype and returns miRNAs related to the phenotype as well as their putative targets based on three different algorithms and a list of validated targets (Laganà et al., 2009). For example, if “Parkinson's disease” is entered, the list of validated miRNAs and targets returned are: has-miR-1:BDNF; has-miR-27b:CYP1B1; has-miR-433:FGF20; miR-124:CYP1B1; has-miR-1:GCH1; has-miR125b:CYP1A1. Also appearing on the list, but without validated target are: has-miR-133b:GCH1. The long list of miRNAs with predicted targets only highlights the difficulty in validation. As the list of miRNAs linked to PD grows, the need to identify their targets becomes increasingly important. The most commonly used tools attempt to identify matches between the miRNA 6–8 nucleotide “seed regions” with the putative target gene 3′ untranslated regions and further employ phylogenetic conservation as an additional criteria (Lall et al., 2006; Ruby et al., 2006; Hammell, 2010). Another approach utilizes experimental data where miRNAs and their targets have been immunopreciptated thus providing experimental support for their predictions (Hammell et al., 2008). Although these tools identify likely candidates, a common issue is their specificity. Toward this end, other methods have been successfully utilized to better predict the putative targets of miRNAs. These include the use of a supervised machine-learning algorithm involving a naive Baysian classifer (Yousef et al., 2007). A naive Bayes classifier is a simple probabilistic classifier with strong independence assumptions. This is well suited for miRNA target identification, where factors such as length of the seed sequence, first nucleotide, and non-seed matches are considered independent of each other. Another intelligent method utilized is self-organizing maps, a semisupervised clustering alogrithm (Heikkinen et al., 2011). Self-organizing maps are artificial neural networks trained using unsupervised learning to generate clusters of data. In the application for identifying miRNA targets in *C. elegans*, all 3′ UTRs in the nematode were utilized for training and building the map, and validated and unvalidated miRNAs where then classified onto the map. The performance of SOM were benchmarked against existing tools and shown to outperform all other algorithms, some significantly by correctly locating the target (classifying) 18/20 experimentally validated miRNAs.

A table summarizing miRNAs, their putative target, and biological process linked to PD is shown as Table 1.

**Table 1:** **Summary of miRNAs linked to Parkinson's disease**.

**miRNA**	**Putative target**	**Biological process or origin**	**References**
miR-133b	Pitx3	dopaminergic neuron development	Kim et al., 2007
miR-9/9^*^, miR-124	Multiple	neuron specification	Yoo et al., 2011
miR-7, miR-153	ASYN	gene regulation	Junn et al., 2009; Doxakis, 2010
miR-7a	Pax6	dopaminergic neuron development	de Chevigny et al., 2012
miR-34b/c	Unknown	PD pathology	Miñones-Moyano et al., 2011
miR-1, miR-22^*^, miR-29	Multiple	blood diagnostics, PD vs. healthy	Margis et al., 2011
miR-16-2^*^, miR-26a2^*^, miR30a	Multiple	blood diagnostics, PD treated vs. PD untreated	Margis et al., 2011
miR-132, miR-134 families	Multiple	blood diagnostics, mild cognitive impairment	Sheinerman et al., 2012
miR-64, miR-65	ptc-1	PD models in *C. elegans*	Asikainen et al., 2010
let-7	Multiple	PD models in *C. elegans*	Asikainen et al., 2010
miR-433	FGF20	neuronal maturation, GWAS	Moore et al., 2005; Wang et al., 2008
miR-1, miR-27b, miR-433, miR-124, miR-1, miR-125b	Multiple	miRO database	Laganà et al., 2009

## Next generation sequencing technologies

The ability to perform massively Sanger sequencing in parallel has given rise to next-generation sequencing technologies (Hutchison, 2007; Tucker et al., 2009). On commercial platforms such Illumina HiSeq, Applied Bioscience Solid, and Life Technologies Ion Torrent semiconductor sequencing, it is possible to obtain up to 600 Gb of sequence data per run. This rate of sequencing has significant implications on the ability to detect miRNAs in PD tissues, identify variants in miRNA sequences or their target sites, and to associate loci that increase the risk for PD. These technologies have been used to both identify novel miRNAs as well as new siRNA classes in model systems (Lee et al., 2006; Ruby et al., 2006; Asikainen et al., 2008). One recently discovered siRNA class termed long non-coding RNA (lncRNA) has >1000 members and nearly half are expressed in specific brain regions in mice (Mercer et al., 2008, 2009). A hint that lncRNAs could influence the course of PD has been suggested by the regulation of a splice variant of PINK1 by a lncRNA (Scheele et al., 2007). While a definitive impact of lncRNAs on PD pathogenic processes remains to be seen, such an impact of lncRNAs has already been reported in Alzheimer's disease (AD), through an antisense transcript of β-site amyloid precursor protein-cleaving enzyme (Faghihi et al., 2008). Other neurodegenerative disorder processes affected by lncRNAs include Huntington's disease (HD), amyotrophic lateral sclerosis (ALS), and spinocerebellar ataxia (Qureshi et al., 2010).

Furthermore, while it has been postulated to utilize miRNA profiles from blood to elucidate associations with PD (Margis et al., 2011), it is now technically feasible and affordable to explore associations between any nucleic acid (DNA or RNA) and PD. These associations would be highly useful as diagnostic biomarkers, as well as for general studies in understanding the etiology of PD. As an example, a pair of miRNA families, miR-132 and miR-134, have been recently studied as plasma markers of future mild cognitive impairment, a harbinger of AD or PD (Sheinerman et al., 2012). Moreover, the use of exosomes, secreted microvesicles available from cell culture media or isolated from the circulation of large animals, as sources of nucleic acid for diagnostics has gained feasibility due to NGS technologies (De Smaele et al., 2010). Next-generation sequencing has also been used to identify methylation differences in the 5′ promoter regions and first intron of ASYN from different brain regions of PD patients and compared to controls (de Boni et al., 2011). Their studies were able to identify differences in methylation during the later stages of Lewy body diseases. A recent report has also utilized next-generation sequencing technologies to identify a rare mutation linked to PD in a Swiss family (Vilariño-Güell et al., 2011). The high throughput of NGS technologies has also lead to a 1000 human genomes project, which has provided better quality human sequence maps with enriched information on SNP location and frequencies, as well as occurrences of indels and gene duplications (1000 Genomes Project Consortium et al., 2012). These data will be highly useful in mapping risk factors for sporadic PD.

Lastly, the long awaited ENCODE project, that had an audacious goal of identifying all the functional elements of the human genome has recently been completed (Ecker et al., 2012). The data is free and available for immediate use. This practically means that every PD-associated gene known to date and those to be identified in the future will have a wealth of basic functional information available concerning it at the genome level. Moreover, with the modENCODE project, parallel orthologous functional genomic data will be available as well from *D. melanogaster* and *C. elegans* (Muers, 2011).

## Conclusions

It is abundantly clear that we are in the early days of understanding, identifying, and realizing the potential of manipulating miRNA levels in the treatment of PD. Novel stem cell methodologies involving the use of miRNAs have given rise to the possibility of replacing lost dopaminergic cells and changing the current paradigms of treatment. Together with next generation sequencing technologies and associated bioinformatics development, the application of knowledge regarding miRNAs and neuronal maturation should lead to exciting new discoveries and eventually effective therapies.

### Conflict of interest statement

The authors declare that the research was conducted in the absence of any commercial or financial relationships that could be construed as a potential conflict of interest.
